# Primary hepatic leiomyoma in a Chinese female patient without underlying disease: a case report

**DOI:** 10.1186/s12893-019-0598-1

**Published:** 2019-10-07

**Authors:** Baoxing Jia, Zhe Jin, Pin Gao, Yahui Liu

**Affiliations:** 1 Department of Hepatobiliary and Pancreatic Surgery, the First Hospital of Jilin University, Changchun, 130021 Jilin China; 2 Department of Breast Surgery, the First Hospital of Jilin University, Changchun, 130021 Jilin China

**Keywords:** Leiomyoma, Liver neoplasm, Primary hepatic leiomyoma, Laparoscopic hepatectomy

## Abstract

**Background:**

Primary hepatic leiomyoma (PHL) is a rare manifestation of tumors in the liver; it is mainly characterized by its origin in the mesenchymal tissue. To date, the mechanisms underlying the pathogenesis of this disease remain unclear, however most reported PHL patients suffer from acquired immunity deficiency syndrome (AIDS) or take immunosuppressive medications after organ transplantation.

**Case presentation:**

In this case report we describe a rare case of PHL in a middle-aged Chinese woman who was asymptomatic with no history of hepatitis or other liver disease. She had no history of immune suppression medication therapy. In view of the benign features of the hepatic lesion, along with our implementation of the respecting the patience choices, a laparoscopic partial hepatectomy of the right lower liver was performed, which appeared to be highly effective and give a good prognosis.

**Conclusions:**

Clinical characteristics of the patient should be compared to previously reported aspects of this disease to reach a clear diagnosis. Moreover, although PHL is extremely rare, it should still be considered a possibility. Surgical intervention is effective in treating this disease.

**Electronic supplementary material:**

The online version of this article (10.1186/s12893-019-0598-1) contains supplementary material, which is available to authorized users.

## Background

Primary hepatic leiomyoma (PHL), a rare manifestation of tumors in the liver [1, 2]; is mainly characterized by its mesenchymal tissue origin in the liver, and no cases of leiomyoma are identified in the gastrointestinal and urinary tracts or elsewhere in the body [3].

To date, the pathogenic mechanisms underlying this disease remain unclear. While it has been postulated that the neoplasia may arise from atypical growth of hepatic vessels and abnormal proliferation of bile ducts [1, 4, 5] this has not been well validated by scientific research.

Since 1926, when the first case of PHL was described, most PHL cases have been reported among patients suffering from acquired immunity deficiency syndrome (AIDS) or AIDS in combination with Epstein-Barr virus (EBV) infection, or taking immunosuppressive medications after organ transplantation [2, 6]. To our knowledge, PHL in immunocompetent patients is extremely rare.

In this case report, we describe an unusual case of PHL in a middle-aged Chinese woman who was asymptomatic with no immunosuppressive disorders, no history of hepatitis or other liver disease, and no history of immunosuppressive medication use. After a successful laparoscopic partial hepatectomy, the patient has an excellent prognosis.

## Case presentation

### Patient description

A 46-year-old Chinese woman consulted a physician in our department for an intrahepatic mass incidentally detected by an abdominal ultrasound (US) during her annual physical examination. After a 7-cm hypoechoic mass in the right lobe of the liver was identified and confirmed, the patient was admitted to our hospital for further investigation and treatment of the hepatic lesion. She reported no immunosuppressive medication use and no history of liver disease or surgery.

### Clinical examination

No mass and discomfort and no signs of other clinical symptoms were detected during the abdominal examination. Hematological and serum biochemical profiles were within normal ranges. Tumor markers including alpha-fetoprotein (AFP), carcinoembryonic (CEA), carbohydrate antigens 199 (CA199), and carbohydrate antigens 125 (CA125) were also normal.

**Fig. 1:**
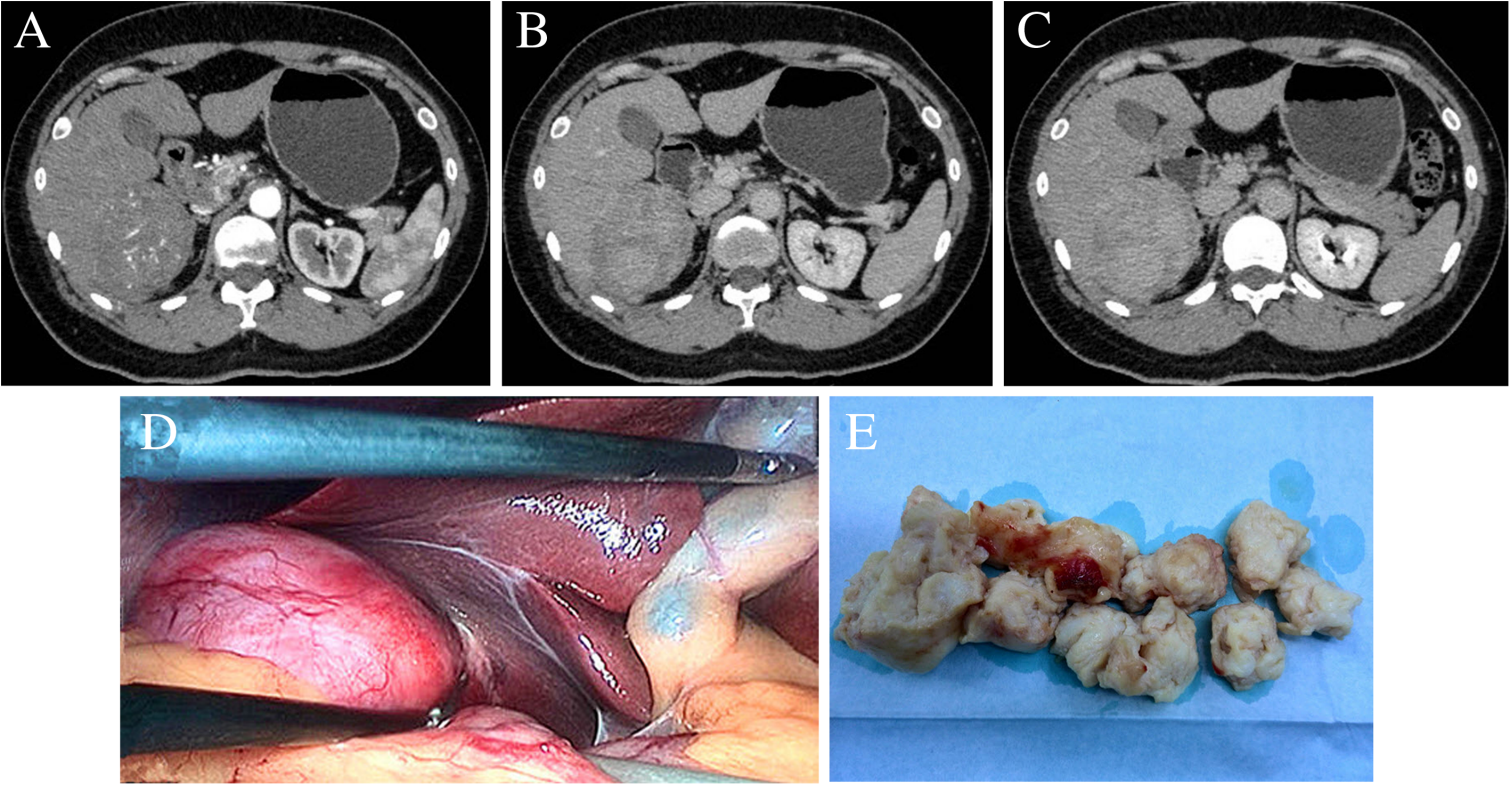
Abdominal CT scan images of the patients. CT images demonstrate (**a**) heterogenous enhancement in the artery phases; **b** progressive enhancement in the portal venous phases; and **c** prolonged enhancement in the balanced phases. **d** An image during the laparoscopic partial hepatectomy. **e** Tumor tissue samples

Further investigation of the hepatic lesion with computed tomography (CT) revealed a slightly hypodense 6.5 × 7.2 cm mass in the right lobe of the liver with heterogenous enhancement of arterial phase, and prolonged enhancement through portal venous phase and the lesion (Fig. 1a, b, c). No other abnormalities were identified in the remaining liver tissue and elsewhere the abdomen.

As the CT imaging was inconclusive, further evaluation with magnetic resonance imaging (MRI) as well as US-guided fine needle aspiration (FNA) biopsy of the tumor for pathological examination was recommended. However, the patient preferred an operation without additional preoperative assessment of the lesion.

### Treatment and outcome

In view of the benign features of the hepatic lesion and in accordance with our patient’s choice of treatment, we carried out laparoscopy (Olympus, Tokyo, Japan). A 12-mm optical trocar at the umbilicus as well as three other trocars was used. Two 12-mm trocars were used through the epigastrium and right upper abdomen. A 5-mm trocar was used through the right abdomen near the anterior axillary line. After a laparoscopic exploration and US, a solid 6.5 cm × 7.0 cm × 7.5 cm mass was clearly located in the segment 6 of the liver and outwardly protruding from the liver surface (Fig. 1d). We decided to perform laparoscopic partial hepatectomy. Hepatic parenchyma was performed using an UltraCision Harmonic Scalpel (Ethicon Endo-Surgery, Cincinnati, OH, USA). The branches of the Glissonian pedicles and hepatic vein within the liver were clipped using a Hem-o-lok® (Teleflex Medical, Morrisville, NC, USA) and a titanium clip (TSCS, Hangzhou, China), and then transected. We put the surgical specimen into a laparoscopic disposable specimen bag (Xueli, Nanchang, China) through the epigastrium incision, and to avoid expanding the incision we cut the specimen into small pieces in the bag before taking them out (Fig. 1e). Finally, the surgical area was carefully examined and a drainage tube was not placed. Seven days after surgery, the patient was discharged from hospital. After a 2-year follow-up, no recurrence or metastasis occurred.

### Final diagnosis

**Fig. 2:**
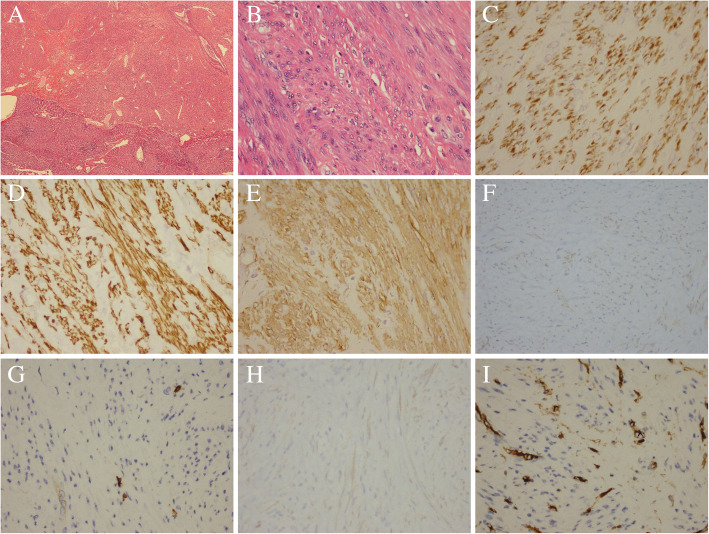
Characterization of primary hepatic leiomyoma staining. **a** Tissue sample staining × 4; **b** tissue sample staining × 40; **c** desmin (+); **d** H-caldesmon (+); **e** SMA (+); **f** Dog-1 (−); **g** CD117 (−); **h**; S-100 (−); **i** CD34 (−)

Postoperative pathological examination revealed a benign smooth muscle tumor derived from the mesenchymal tissue of the liver with clearly visible margins and with no evidence of necrosis or tumor invasion (Fig. 2a, b). Immunohistochemistry (IH) showed strong reactivity for smooth muscle actin (SMA) (+), desmin (+), and H-caldesmon (+) (Fig. 2c, d, e), but not for Dog-1 (−), CD117 (−), S-100 (−), and CD34 (−) (Fig. 2f, g, h, i). According to the diagnostic criteria for PHL, the patient was finally diagnosed with PHL.

## Discussion and conclusions

Since 1926 when the first case of PHL was described, just 22 immunocompetent cases have been reported in the medical literature (Table 1). Here we reported the 23rd case. The average age was 48.17 years and the male to female ratio was 6:17. The mass size ranged from 3 cm to 30 cm. Thirteen cases were found in the right lobe of liver, eight cases in the left lobe, and two cases in the caudate lobe.

**Table 1:** PHL without immune-compromised cases in medical literature

	Author	Year	Age/Sex	Symptoms	Location/Size (cm)	Treatment
1	Demel [7]	1926	42/F	RUQ pain	RL/12	Laparotomy
2	Rios-Dalenz [8]	1965	87/F	RUQ pain	LLL/NS	Autopsy
3	Ishak et al. [9]	1975	64/M	Abdominal mass	RL/NS	Laparotomy
4	Hawkins et al. [3]	1980	66/M	Abdominal mass	LL/13	Left hepatectomy
5	Hollands et al. [5]	1989	17/M	UA pain	LL/9	Left hepatectomy
6	Herzberg et al. [10]	1990	30/F	RUQ pain	RL/19	Partial right hepatectomy
7	Bartoli et al. [11]	1991	34/F	None	RL/NS	Right hepatectomy
8	Reinertson et al. [12]	1992	32/F	RUQ pain	LL/10	Left hepatectomy
9	Yanase et al. [13]	1999	59/F	Liver dysfunction	RL/13	Right hepatectomy
10	Mesenas et al. [14]	2000	59/M	None	RL/3.6	Segmentectomy (S5)
11	Belli et al. [4]	2001	67/F	Abdominal mass	RL/30	Right extended resection
12	Kanazawa et al. [15]	2002	31/M	None	LLL/3.5	LLL resection
13	Beuzen et al. [16]	2004	36/F	RUQ pain	LLL/5	LLL resection
14	Imasato et al. [17]	2005	61/F	None	CL/4.5	Right hepatectomy
15	Urizono et al. [18]	2006	71/M	None	CL/3	Partial hepatectomy
16	Marin et al. [19]	2008	64/F	None	RL/3	Right hepatectomy
17	Sousa et al. [20]	2009	61/F	Dyspepsia	LLL/9.5	Left hepatectomy
18	Kalil et al. [21]	2009	44/F	Abdominal mass	RL/7	Atypical resection
19	Santos et al. [22]	2011	28/F	None	RL/5.5	Segmentectomy
20	Perini et al. [23]	2012	45/F	RUQ pain	RL/16.5	Segmentectomy
21	Vyas et al. [24]	2015	20/F	UA pain	LLL/8	LLLL resection
22	Navarro et al. [25]	2015	44/F	None	RL/NS	Segmentectomy (S5,7,8)

*RUQ* Right upper quadrant, *RL* Right lobe, *LLL* Left lateral lobe, *NS* Not stated, *LL* Left lobe;

*UA* Upper abdomen, *CL* Caudate lobe, *LLLL* Laparoscopic left lateral lobe

A PHL diagnosis needs to satisfy the following criteria: (1) the tumor originates from the hepatic mesenchymal tissue; and (2) there are no primary tumors elsewhere in the body. However, despite this clear criteria for identifying this rare type of intrahepatic tumor, a successful preoperative diagnosis of PHL is challenging, mainly due to imaging features being similar with other benign hepatic tumors as well as a lack of specific characteristics to guide physical and laboratory examinations [5, 8, 23].

Several common imaging patterns have been identified in PHL patients. On US scans, PHL appears as heterogenous hypoechoic nodules [16, 18, 20]; a finding also seen in our case. In CT imaging, hypodense lesions are widely reported with marked enhancement in the arterial and portal venous phases, occasionally in the peripheral phase, and with prolonged enhancement in the equilibrium phase [5, 16, 20]. On MRI, the PHL usually presents a lower signal in T1-weighted images and a higher signal in the T2-weighted images [2, 18, 23]. In addition, lesion characteristics (irregular margins) on hepatic angiography have been reported in a few PHL cases [15, 18]. Non-invasive, preoperative imaging patterns on US, CT, MRI, and angiography can not define PHL.

Of the reported PHL cases in the literature, Sousa and colleagues achieved an accurate diagnosis of PHL in a healthy middle-aged woman by undertaking an imaging-guided fine needle aspiration (FNA) and a 18G tru-cut liver biopsy of the tumor tissue [20]. Sadler and colleagues reported two cases with preoperative diagnosis on liver biopsy: one case with mesenchymal mixed tumor of the liver but an accurate diagnosis could be not reached in the other case [26]. In our patient, like most reported cases, a non-invasive, preoperative diagnosis with imaging features was inconclusive.

After a postoperative pathological examination was carried out on the biopsy specimen, the pathological features were noteworthy. A benign smooth muscle tumor containing the mesenchymal tissue with clear margins was visualized. Positive staining for SMA, which is observed in most reported PHL cases and is a hallmark of PHL, was confirmed in this case. Moreover, positive staining for desmin and H-caldesmon, as observed in some reported PHL cases [14], was also noticed in our patient, whereas vimentin was negatively expressed in the tumor tissue (also reported in some PHL cases). In addition, negative staining for Dog-1 and CD117 distinguished PHL from the gastrointestinal stromal tumors (GIST), while negative reactivity for S-100 ruled out leiomyoma of the central nervous system and CD34 excluded vascular leiomyoma. The patient thus fulfilled the two criteria and was eventually diagnosed with PHL.

In this case, given the benign features of the hepatic lesion, which usually signifies treatment with surgery [27] as well as our respect for the patient’s preferred choice of treatment, we performed a laparoscopic partial hepatectomy of the right lower liver [24]. The procedure appeared to be highly effective in this case, and our patient has a good prognosis and is expected to have no recurrence in the long term.

In conclusion, the imaging and pathological features of the patient should be added to previously reported aspects of this disease. Moreover, although PHL is extremely rare, it should still be considered a possibility. Surgical intervention is effective in treating PHL.

## Additional file

Additional file 1: Data of Patients. (XLSX 10 kb)

## Data Availability

All data generated or analysed during this study are included in this published article and its supplementary information files (Additional file 1).

## Abbreviations

AFP: Alpha-Fetoprotein
AIDS: Acquired Immunity Deficiency Syndrome
CA125: Carbohydrate Antigens 125
CA199: Carbohydrate Antigens 199
CEA: Carcinoembryonic
CT: Computed Tomography
EBV: Epstein-Barr Virus
FNA: Fine Needle Aspiration
GIST: Gastrointestinal Stromal Tumors
IH: Immunohistochemistry
MRI: Magnetic Resonance Imaging
PHL: Primary Hepatic Leiomyoma
SMA: Smooth Muscle Actin
UA: Abdominal Ultrasound
